# Experimental and Numerical Study of a Thermal Expansion Gyroscope for Different Gases †

**DOI:** 10.3390/s19020360

**Published:** 2019-01-17

**Authors:** Guillaume Kock, Philippe Combette, Marwan Tedjini, Markus Schneider, Caroline Gauthier-Blum, Alain Giani

**Affiliations:** 1 Institut d’Électronique et des Systèmes, Université de Montpellier, IES, F-34090 Montpellier, France; philippe.combette@umontpellier.fr (P.C.); marwan.tedjini@ies.univ-montp2.fr (M.T.); giani@ies.univ-montp2.fr (A.G.); 2 The French-German Research Institute of Saint-Louis, F-68301 Saint Louis CEDEX, France; MARKUS.SCHNEIDER@isl.eu (M.S.); CAROLINE.GAUTHIER-BLUM@isl.eu (C.G.-B.)

**Keywords:** gas thermal gyroscope, MEMS, thermal expansion, shock resistance, numerical simulation

## Abstract

A new single-axis gas thermal gyroscope without proof mass is presented in this paper. The device was designed, manufactured and experimentally characterized. The obtained results were compared to numerical simulation. The working principle of the gyroscope is based on the deflection of a laminar gas flow caused by the Coriolis effect. A bidirectional hot air flow is generated by alternating activation of two suspended resistive micro-heaters. The heated gas is encapsulated in a semi-open cavity and the gas expands primarily inside the cavity. The thermal expansion gyroscope has a simple structure. Indeed, the device is composed of a micromachined cavity on which three bridges are suspended. The central bridge is electrically separated into two segments enabling to set up two heaters which may be supplied independently from each other. The two other bridges, placed symmetrically on each side of the central bridge, are equipped with temperature detectors which measure variations in gas temperature. The differential temperature depends on the rotational velocity applied to the system. Various parameters such as the heating duty cycle, the type of the gas and the power injected into the heaters have been studied to define the optimal working conditions required to obtain the highest level of sensitivity over a measurement range of around 1000°/s. The robustness of the device has also been tested and validated for a shock resistance of 10,000 g for a duration of 400 µs.

## 1. Introduction

A gyroscope is an inertial sensor used for precise measurement of the angular velocity of a moving object. Silicon-based, micro-structured gyroscopes are among the most commonly manufactured sensors comprising pressure sensors and accelerometers. In general, the interest shown for this type of sensor may be explained by the fact that their integration coupled with that of the data processing electronic circuitry on the same substrate enables a considerable size reduction. It is for this very reason that it is common in the industrial sector to integrate a large number of devices on the same wafer, thus enabling large-scale production with consequent reduction in the manufacturing cost for individual micro-sensor and electronic sub-assemblies. Micro-structured gyroscopes have therefore been extensively studied and developed for decades and now, with the emergence of Micro-Electro-Mechanical System (MEMS) technology, these sensors have gradually matured. Thanks to their reduced size, possibility of mass production and low cost, these gyroscopes now play a key role in industrial, civil and military applications. In fact, gyrometric sensors are widely used in numerous applications such as inertial navigation and missile guidance systems, as well as smartphones, displacement monitoring equipment, virtual reality devices, image stabilization systems for digital video cameras or even vehicle safety systems [1,2].

Most MEMS gyroscopes use vibrating mechanical elements to measure the angular velocity. The working principle is based on the Coriolis sinusoidal force induced by the angular velocity which provokes coupling of the energy between two modes of vibration on a proof mass. The primary mode of vibration is referred to as the excitation mode which enables the mass to vibrate in a linear direction. It is, in most cases, generated by an electrostatic or electromagnetic force. The secondary mode of vibration is referred to as detection mode. The Coriolis effect induced by the rotation results in a transfer of energy to the detection mode which is proportional to the angular velocity. Displacement of the internal structures of the gyroscope caused by the vibrations in the secondary mode may be detected using capacitive, piezoelectric or piezoresistive means [3,4,5]. Nevertheless, the presence of a proof mass and moving mechanical parts inside vibratory gyroscopes result in intrinsic problems such as potential operating fragility in the long term, as well as low resistance to mechanical shocks [6,7].

On the other hand, fluidic gyroscopes use liquid (water, electro-conjugate fluid corresponding to a dielectric liquid) [8,9] or gas (air, sulphur hexafluoride: SF_6_) [10,11] as mobile and sensitive elements instead of the proof mass thus avoiding the use of mechanical moving parts with the associated breakage problems. In spite of their simple structure, low cost and high shock resistance, these fluidic gyroscopes are confronted with challenges still to be overcome in terms of sensitivity, bandwidth, measurement range, bias drift and so forth. [11,12].

In the particular case of fluidic gyroscopes, there are two types of sensors referred to as jet flow gyroscopes and thermal gas gyroscopes. Jet flow gyroscopes use a laminar gas stream generated by a micropump [13] or by corona discharge ion wind [14], whereas thermal gas gyroscopes use a laminar gas stream produced by thermal convection or thermal expansion. The thermal gas gyroscope was first proposed by Ding, Zhu and Yang in 2001 [15], then the first prototype of a free thermal convection gyroscope type MEMS was presented by Zhu, Su and Ding in 2005 [16].

In the case of the gyroscope based on thermal expansion, the first prototype was presented by Bahari, Feng and Leung in 2012 [7]. The structure of the device is composed of two resistive temperature detectors placed on each side of two micro-heaters. The board-mounted heaters and temperature detectors are suspended over a cavity etched into a silicon substrate. Other designs have been proposed for this sensor and the studies carried out concentrated in particular on the suppression of cross-coupling with acceleration [17,18,19,20]. The design proposed by Cai and al. consists of three heaters, four thermistors and a sealed micro chamber, which is filled with gas medium [18]. The simultaneous measurements of 1-axis angular rate and 1-axis acceleration in one chip have been demonstrated experimentally. They have shown that there is a low coupling effect between acceleration and rotational velocity via using thermal expansion flow. Wang and al. [19] have presented a multi-axis gas sensor can achieve the simultaneous detection of 1-axis angular rate with a nonlinearity of 3.87% within the input range of ±2160°/s and 2-axis acceleration using one chamber. The sensing chip is made of 4 heaters placed at the centre and surrounded by 8 identical thermistors. Liu and Zhu [20] proposed a thermal gas Micromachined Inertial Measurement Unit based on thermal expansion, which measures 3-axis angular rates and 3-axis accelerations using only 3 thermal gas inertial sensors and each are able to measures 1-axis angular rate and 1-axis acceleration simultaneously in one chip. The effectiveness of a compensation method based on a neural network for reduced the measurement errors of 3-axis angular rates and 3-axis acceleration in the measuring range of ±600°/s and the acceleration range of ±1 g, respectively, have been demonstrated experimentally.

In this paper, we present an experimental study of the behaviour of a thermal gas gyroscope based on the thermal expansion principle. The aim of this study is to examine various parameters such as the heating duty cycle, type of gas and power injected into heaters, for the purpose of, for example, optimizing the sensitivity over a measurement range of a few rotations per second. The influence of the type of gas into the cavity with different densities such as dinitrogen (N_2_), argon (Ar), krypton (Kr) and sulphur hexafluoride (SF_6_) has been studied. The robustness of the device has also been tested with a shock test. Furthermore, a numerical simulation model has been developed to analyse the influence of parameter sweeps. Some results refer to previously published work [21].

## 2. Working Principle

**Figure 1:**
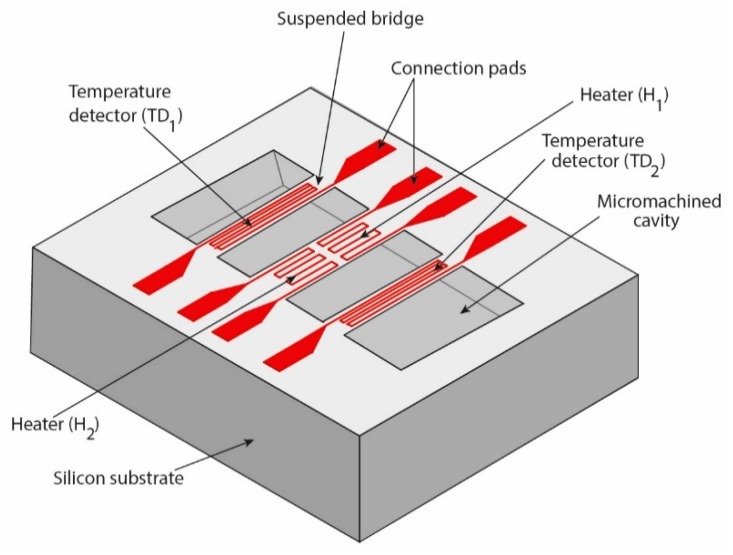
Thermal expansion gyroscope design.

The measurement principle of the gyroscope based on a variation in thermal exchanges by expansion is that of deflection by Coriolis force ($\vec{F_{C}}=2m\vec{v}⋀\vec{\Omega }$) of a hot gas within a cavity. To achieve this, the thermal gyroscope is composed of two heaters and two temperature detectors suspended over a cavity (Figure 1).

**Figure 2:**
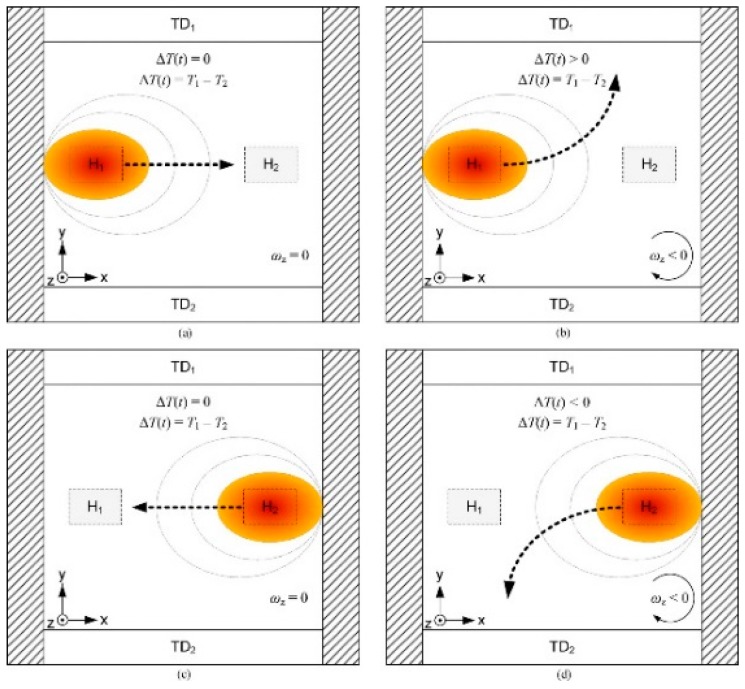
Working principle of a thermal gyroscope [11]. (**a**) rotation off and H_1_ on, (**b**) rotation on and H1 on, (**c**) rotation off and H_2_ on, (**d**) rotation on and H_2_ on.

The flow generated by thermal expansion is produced by alternately heating and cooling two heat sources, thus creating a bidirectional gas flow between two zones of the cavity [11]. If there is no component associated with linear acceleration in drawing X-Y, then the gyroscope will function as shown (Figure 2). Any Coriolis acceleration linked with system rotation will deflect the gas flow and produce an asymmetric temperature profile, which in turn will result in a temperature difference between both detectors ΔT = TD_1_ − TD_2_ (Figure 2). For example, if axis rotation is clockwise when heater H_1_ is activated, then the gas flow is deflected towards TD_1_ and therefore ΔT > 0 (Figure 2b). Whereas, when the heat source H_2_ is activated, the gas flow is deflected towards TD_2_ and therefore ΔT < 0 (Figure 2d). With no Z-axis rotation, the gas flow is not deflected and the latter is symmetric in relation to detectors and therefore ΔT = 0 (Figure 2a,c). Extraction of the gyroscopic measurement requires use of a synchronous detection method which will allow “rectification” of the signal in order to obtain a single output signal proportional to the rotational velocity [11]. 

## 3. Design and Experimental Process

### 3.1. Design and Manufacturing Process

**Figure 3:**
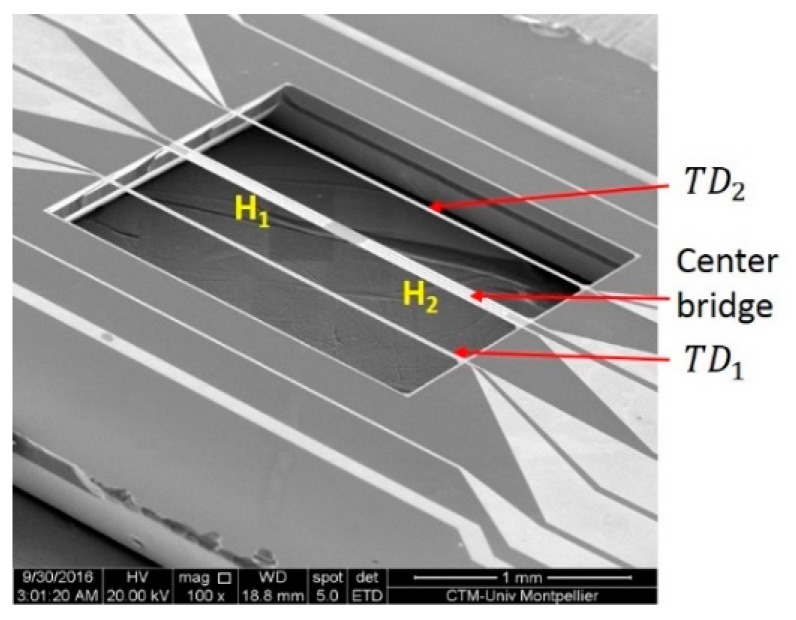
SEM image of micromachined cavity with three suspended bridge.

Production of this microsensor comprises a series of technological stages on silicon, a detailed description of which is provided in reference [22]. The initial stage consists of applying an insulating layer composed of Silicon Nitride (SiN_x_) with a thickness of 500 nm. This stage is required to enable electrical separation of the silicon substrate, which is a semi-conductor and the Platinum (Pt), which is metal. Furthermore, this layer serves as a thermal insulator. Indeed, placing the Pt, which is a good conductor, on a SiN_x_ substrate, which has a lower conductivity capacity, will avoid the transfer of heat via the metal tracks and excessive heating of the substrate. The second stage consists of applying a 300 nm thin Pt layer on the SiN_x_. Thanks to its thermo-resistive property, this Pt layer is used as an active element for both the heaters and detectors. Nevertheless, this layer cannot be applied directly onto the SiN_x_ as the Pt will not adhere to this layer. An adhesive layer of a few tenths of a nanometre must be placed between the two in order for the Pt to adhere to the substrate. This layer is composed either of chromium oxide or zirconium oxide which will also prevent diffusion of the metal (chromium or zirconium) into the Pt layer over time. Two photolithographic and Reactive Ion Etching steps are used to define metal patterns and to create openings in the SiN_x_ to access the silicon substrate. The final manufacturing stage is to create a micromachined cavity (2000 × 2000 × 400 µm) by means of chemical etching using potassium hydroxide. This stage also liberates the suspended bridges (Figure 3).

**Figure 4:**
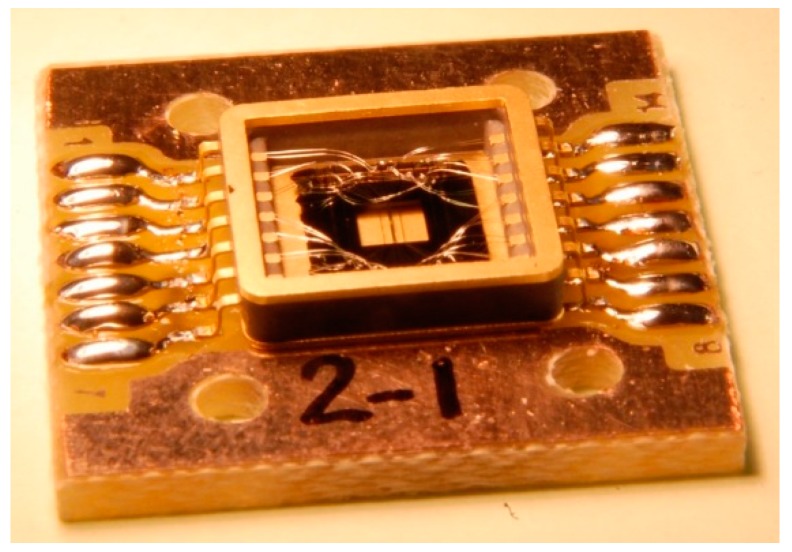
Photo of the gyroscope mounted on a PCB holder.

The sensor is then mounted into a package on an adapted PCB support whereby each heater is micro-soldered to copper tracks (Figure 4). To conclude, the package is covered with a metal casing with overall dimensions of 8 × 8 × 2 mm.

### 3.2. Experimental Setup

**Figure 5:**
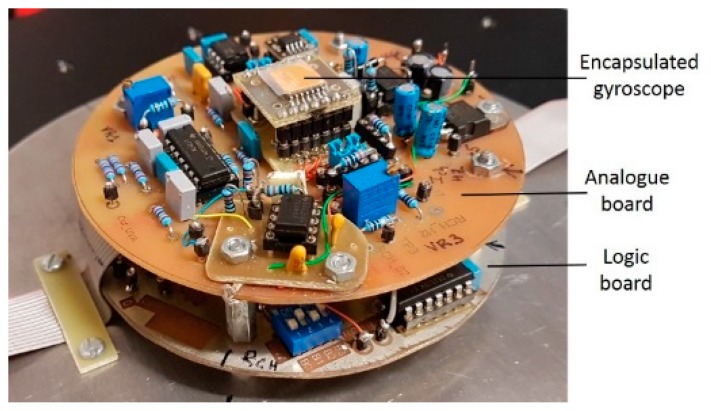
Photo of an encapsulated gyroscope and its associated electronics: an analogue board (upper part) and a logic board (lower part).

To carry out the gyroscopic measurement, a readout electronic has also been developed based on the works proposed by Bahari and al. [11]. To achieve this, two boards have been produced for the supply, output and processing signals, one of which, referred to as the logic board, is placed under the other referred to as the analogue board (Figure 5). The logic board is used to generate and control the numeric signals which alternate the heating process between the two heaters. To do so, a window signal is used with a duty cycle of 25 to 50% in steps of 6.25%, controlled by a set of four switches. The gyroscope is placed on the analogue board which also contains control circuits and signal processing used to extract the gyroscopic measurement (Figure 5). Initial studies were carried out with a cavity filled with dinitrogen (N_2_) to be replaced later by another gas.

**Figure 6:**
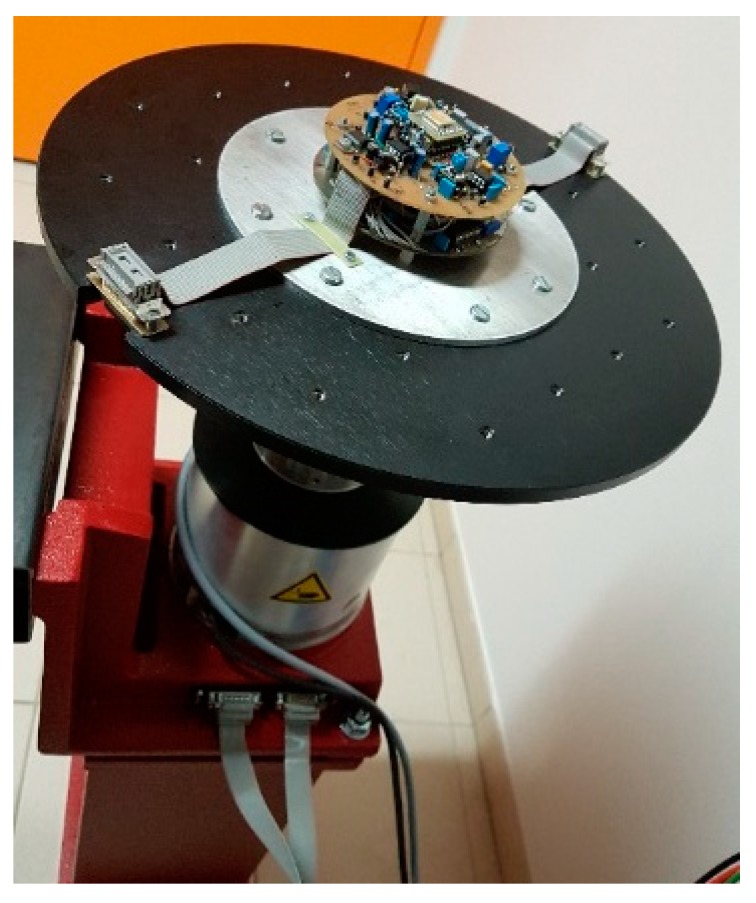
Photo of the device mounted on the rate table.

Both boards and the gyroscope are then mounted in the centre of a single-axis, precision rotating rate table of the brand Acutronic [23]. This apparatus is used exclusively as a test bench for trials and calibration of inertial navigation systems, sensors and other inertial components. It is equipped with a direct-drive brushless motor providing high-torque rotation at a controlled speed over a wide operating range. Indeed, this apparatus may be used in an operating range of ±3000°/s with a resolution of 0.001°/s and a stability of 0.001% for a maximum table load of 20 kg. The rate table is equipped with a 30-track collector ring, the cables for which are terminated with 2 D-Sub connectors on the table top and the corresponding plug-in connectors at the base of table (Figure 6). This setup provides for safe working conditions as well as easy supply of the device and recovery of signals during table rotation.

### 3.3. Numerical Design

**Figure 7:**
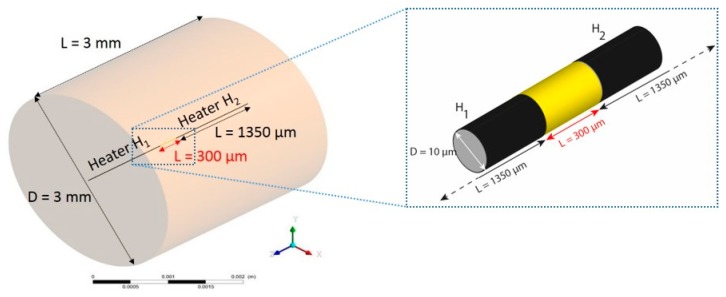
Design and dimension of geometry chosen for numerical studies.

The cavity chosen is illustrated by a cylinder with a length of 3 mm and diameter of 3 mm (Figure 7). These dimensions are a volumetric average of those in the experimental prototype. The meshing used compared to a parallelepipedal enclosure is far simpler. It enables use of a structured meshing and thus avoid angles, which reduces the number of meshing nodes and consequently offers a shorter calculation time and the convergence of simulations is much faster. A central cylindrical bridge with a diameter of 10 µm and a length equal to that of the cavity is shown. This bridge is divided into three segments comprising both heaters (sources left: H_1_ and right: H_2_) separated by a non-heated central element of 300 µm (Figure 7). These choices were determined but not exclusively, by future bridge technologies to be adopted.

Displacement of the gas, dinitrogen in our case, is due to injection of electrical power alternating between the two sources located on the central bridge. Detection is achieved with virtual detectors placed at a distance of 300 µm on each side of the central bridge along the X-axis and which have a length corresponding to that of the cavity (3 mm). This choice was also arrived at by experimental trials. The system is rotated in the Y-axis. Various numerical studies were carried out using Ansys Fluent CFD software.

**Figure 8:**
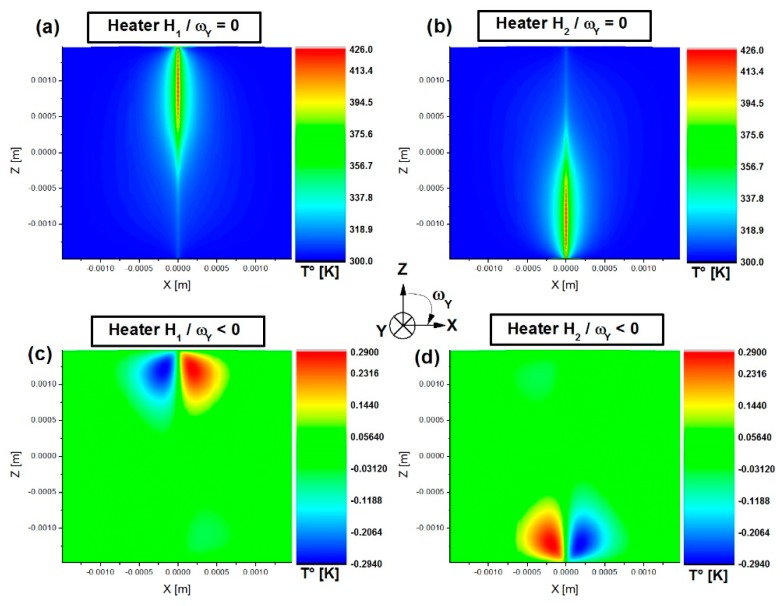
Temperature mapping (K): (**a**) Heater H_1_ activated for ω_Y_ = 0; (**b**) Heater H_2_ activated for ω_Y_ = 0; (**c**) Heater H_1_ activated for temperature difference between ω_Y_ = 300°/s and ω_Y_ = 0; (**d**) Heater H_2_ activated for temperature difference between ω_Y_ = 300°/s and ω_Y_ = 0.

Figure 8 provides an example of a simulation for injection of electrical power over a time period of 7.9 ms corresponding to a duty cycle of 25%. This figure represents the gas temperature (K) mapping for source H_1_ (Figure 8a) and for source H_2_ (Figure 8b) in the case of a system not subjected to rotation. Figure 8c illustrates the same temperature mapping but with the difference between the system subjected to rotation (ω_Y_ = 300°/s) and the stationary system when H_1_ is activated, whereas Figure 8d shows the case with activation of H_2_. The latter two mappings show that the hot and cold zones are not located on the same side depending on which source is activated. Indeed, this is due to the flow direction which is inverted each time source activation is alternated resulting in displacement of the gas. This is coherent with the working principle presented in Part II.

## 4. Results and Discussions

### 4.1. Sensor Linearity and Sensitivity

#### 4.1.1. Numerical Part

**Figure 9:**
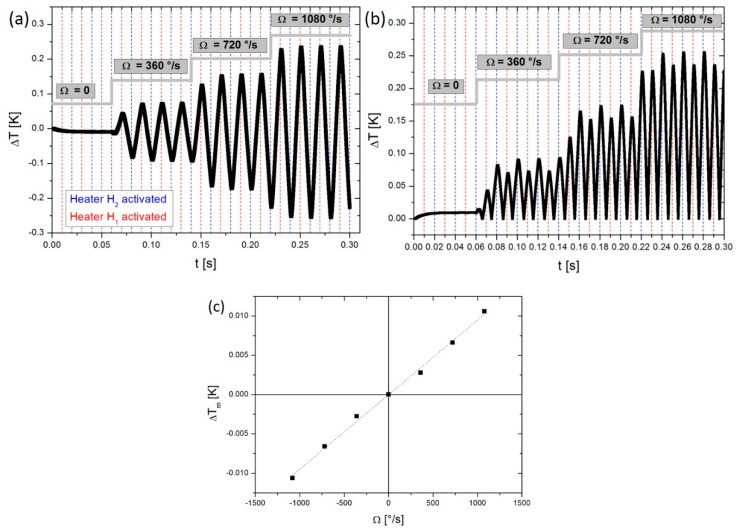
Extraction of the output system by processing the detection signals: (**a**) ΔT for different rotational velocities; (**b**) “rectification” of the previous signal; (**c**) output signal according to rotational velocities.

To check validity of the device’s reaction to a gyroscopic phenomenon, signals generated by numerical simulation and more specifically those indicating sensor sensitivity, have been analysed. These are then compared with those presented in the works of A. Leung et al. [24,25]. The alternating heating sequence is achieved by injecting power alternatively into one heater and then the other with a duty cycle of 50%. Figure 9a illustrates the temperature difference between the two virtual detectors which corresponds to the detection sensitivity image, in this case located at 300 µm on each side of the central bridge, obtained for various rotational velocities.

One notes that the value of this variable changes its sign with each half period of heating, thus confirming the displacement of gas due to thermal expansion. The output signal is then “rectified” every half-cycle in a synchronous manner with alternated heating, as may be seen in Figure 9b. Finally, Figure 9c shows the average value of the previous signal which enables us to obtain the average temperature difference in accordance with the rotational velocity. The latter illustrates the gyroscopic output of the device which is linear in relation to the rotational velocity. The sensitivity obtained is around 10^−2^ mK/(°/s) with a non-linearity of 0.1%.

#### 4.1.2. Experimental Part

To begin with, in order to ensure the output was not influenced by physical phenomena other than the thermal expansion, the system was subjected to a rotation without supplying heaters but by feeding detection circuits. No output signal variation was noted. This fact confirms that gas displacement and the associated output signal, were a result of thermal expansion.

**Figure 10:**
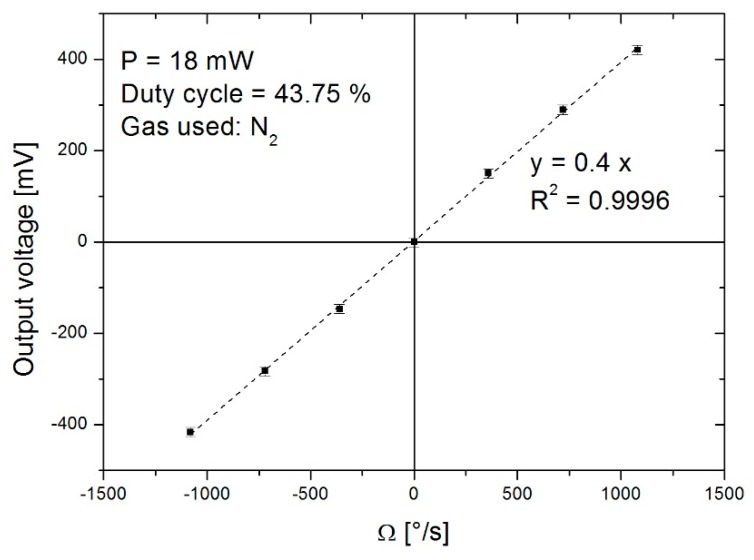
Output system vs rotational velocity for a duty cycle of 43.75% with a frequency of 32 Hz and heater power of 18 mW.

For this study, parameters used are a duty cycle of 43.75%, a detection and heating current of 1.5 mA and 8.5 mA (P = 18 mW), which corresponds to resistors temperature of around 310 K and 490 K. These parameters are applied to the device and then checked before beginning rotation. The system response with a rotational velocity of up to ±1080°/s is plotted in Figure 10. These results were obtained using an electronic gain amplification of 81820. The gyroscope presents a sensitivity of 0.4 mV/(°/s) with a non-linearity of around 0.1% for the measuring range considered, which is coherent with the works presented by Bahari et al. [11].

In our case, this measuring range is the maximum applicable value with the current system. Indeed, the readout electronic size, the various fastening elements and associated wiring do not allow us to apply a higher rotational velocity. However, there would appear to be no doubt that the measurement range should be higher than that presently applied. To achieve this, we hope to be able to produce far more integrated and compact readout electronic in the future capable of withstanding far higher rotational velocities.

By comparing the Figure 9c and the Figure 10, we can see the good correlation between the simulation and the experiment with a linear behaviour of the sensor over a measurement range of ±1080°/s.

### 4.2. Study of Heating Power on the Sensitivity with N_2_ Medium

Increasing the heating power logically increases the temperature in heaters, which in turn raises the temperature of the gas. The influence of heating power on sensitivity is studied.

**Figure 11:**
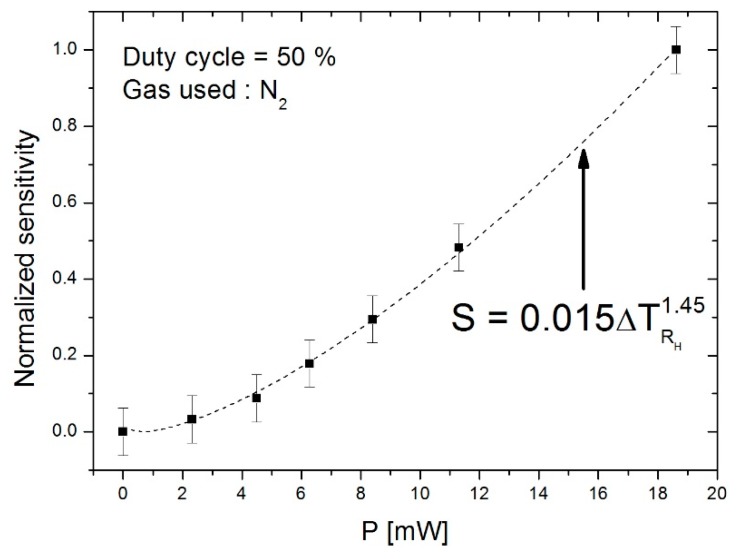
Evolution of the normalized sensitivity as a function of the power injected into heaters for a duty cycle of 50% with a frequency of 32 Hz.

Results of this operation are shown in Figure 11, which illustrates the relative sensitivity as a function of the power injected into heaters for a duty cycle of 50%. One notes that an increase in the power injected into heaters improves sensitivity of the device. Indeed, a higher level of heating power increases the gas temperature, which, once deflected, subsequently increases the temperature at the detectors, the consequence of which is a higher temperature difference between the detectors.

The best interpolation graph is given with an exponential power close to 1.45 between the heating power and the sensitivity. We will therefore have a behavioural model whereby the sensor sensitivity will be proportional to the increase in heater temperature with a power close to 1.45. In order to obtain a high level of sensitivity, it would therefore be of interest to use a higher heating power. However, above 18 mW, we approach the power tolerance limit of the suspended heaters. For this reason, the increase in power injected is limited.

### 4.3. Influence of the Type of the Gas on Sensitivity

**Figure 12:**
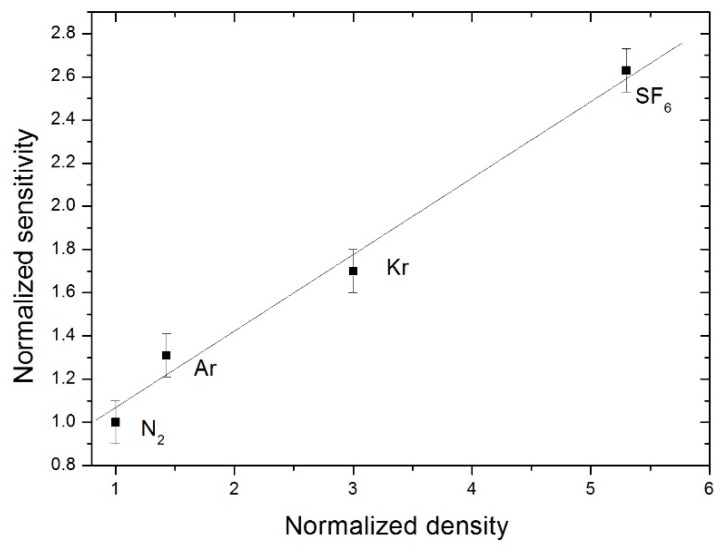
Evolution of the normalized sensitivity vs normalized density of the encapsulated gas for a duty cycle of 37.5% with a frequency of 32 Hz and 18mW heater power.

As force is proportional to mass, in the case of Coriolis force, one may assume that the denser the gas, the higher the force will be and subsequently gas deflection as well as sensitivity will increase as a result. It is for this reason that the sensor sensitivity has been designed with gases of various densities encapsulated inside the cavity. Results are presented in Figure 12, indicating the sensitivity as a function of the density, both of which are normalized for four different gases: dinitrogen (N_2_), argon (Ar), krypton (Kr) and sulphur hexafluoride (SF_6_). The present study was carried out with a heating power of 18 mW and a duty cycle of 37.5%. The choice of this duty cycle will be explained elsewhere in this document. One notes that the higher the gas density, the higher the sensitivity will be in accordance with a behavioural law similar to a linear law.

**Figure 13:**
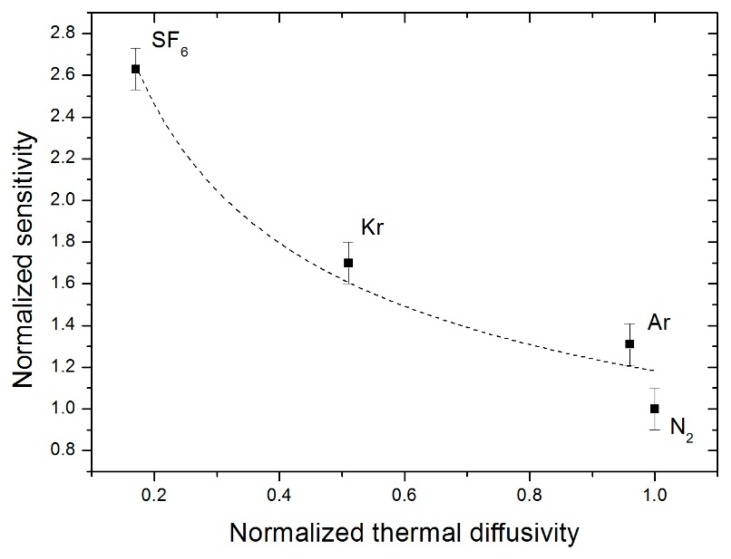
Evolution of the normalized sensitivity as a function of the normalized thermal diffusivity for a duty cycle of 50% with a frequency of 32 Hz and 18 mW heater power.

Conversely, one notes in Figure 13 that the greater the increase in thermal diffusivity, the lower sensitivity will be. In the present study, two factors must be considered depending on the type of the gas. Indeed, the first is the thermal conductivity which describes the capacity of the gas to conduct heat in terms of amplitude, that is, the higher it is, the higher thermal transfer will be [26,27]. Secondly, there is the thermal diffusivity which describes the capacity to transfer heat in terms of speed within a studied body [26,27]. Therefore, in the case of gasses such as argon and dinitrogen, which have high levels of thermal conductivity and diffusivity, the heat transferred by the heater will diffuse farther into the cavity, considering the temperature in the cavity. Consequently, the temperature difference between the two detectors generated by the asymmetric flow will be lower for these two gasses. For this reason, a gas such as SF_6_, which offers a lower level of thermal conductivity and diffusivity than the latter gasses, will allow us to obtain a higher temperature difference and consequently a high level of sensitivity. 

### 4.4. Response to the Heaters Duty Cycle for Different Gases

The heating duty cycle is linked to the heater activation time allowing injection of electrical power into each heater. The duty cycle may be modulated between 25 and 50%, in steps of 6.25%. Above 50%, both heaters are activated at the same time which is not compatible with the desired function as the two flows generated will be in opposing directions. 

**Figure 14:**
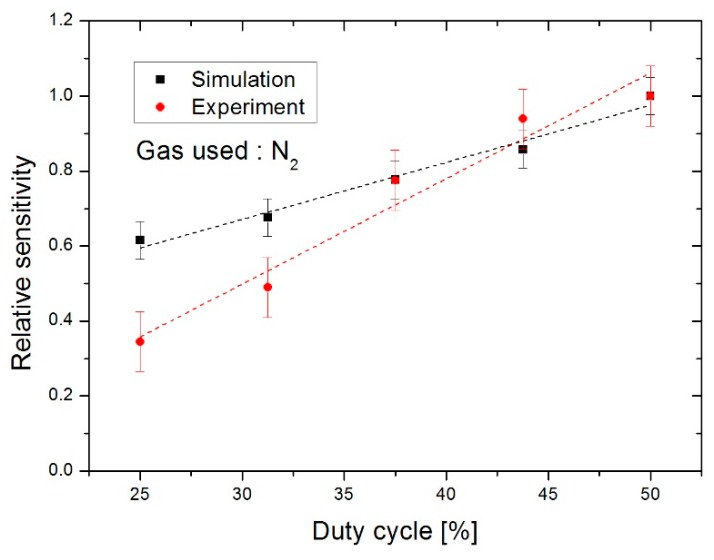
Evolution of the relative sensitivity as a function of the duty cycle.

By comparing experimental measurements with numerical simulation results to ascertain the influence of the duty cycle, one notes that evolution of the sensitivity is relatively identical in both cases, as can be seen in Figure 14. An increase in duty cycle provides an increase in sensitivity. Consequently, under these conditions and using dinitrogen gas, it is of greater interest to work with a duty cycle of 50%, in order to obtain the highest level of sensitivity. 

**Figure 15:**
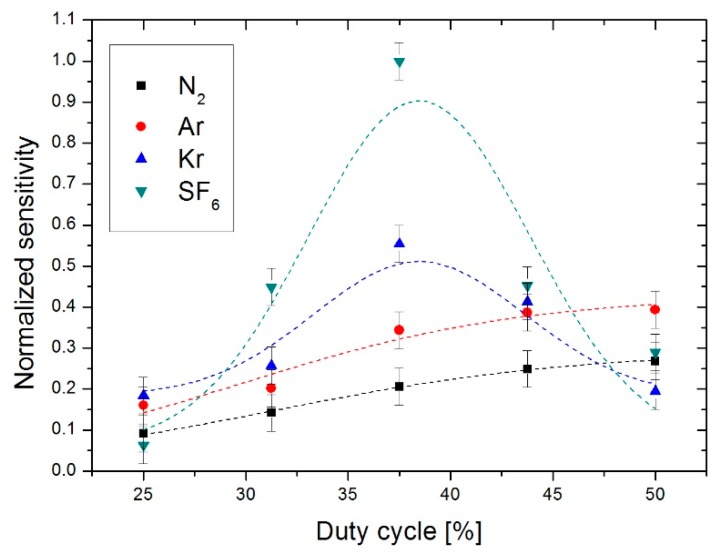
Evolution of the normalized sensitivity according to the type of the gas as a function of the duty cycle at 18 mW heater power and 32 Hz heater switching frequency.

However, this is not valid for all gases. Indeed, the same study was carried out experimentally with the different gases presented above according to the duty cycle. As seen in Figure 15, one notes that in the case of gases such as dinitrogen and argon, the optimal duty cycle, that is, the ratio used to obtain the highest level of sensitivity, is 50%. Whereas, in the case of gases denser than the latter such as krypton and sulphur hexafluoride, the optimal duty cycle is 37.5%. This is confirmed by the works of Bahari and al. [11] which illustrates that the optimal duty cycle is 37.5% for a similar study carried out exclusively using SF_6_.

Therefore, depending on the type of the gas used, the duty cycle for the heaters shows, for example, an optimal range between 50% and 37.5% for dinitrogen and sulphur hexafluoride, respectively.

### 4.5. Numerical Determination of the Optimal Position of Detectors with N_2_ Medium

**Figure 16:**
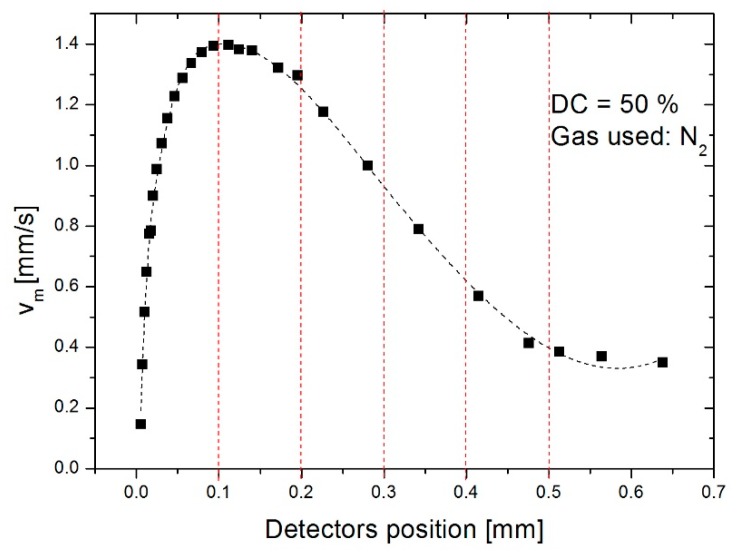
Velocity gradient between the central bridge and position of detectors studied.

Thermal expansion is a phenomenon which generates movement of gas at a relatively low velocity (in the order of mm/s) along a gradient in the cavity. Figure 16 shows this gradient as a function of the position of the detectors studied between 100 and 500 µm, in steps of 100 µm, in relation to the symmetrical axis (central bridge). One notes that the average gas flow velocity at a maximum with the detector at 100 µm and then it decreases progressively. 

**Figure 17:**
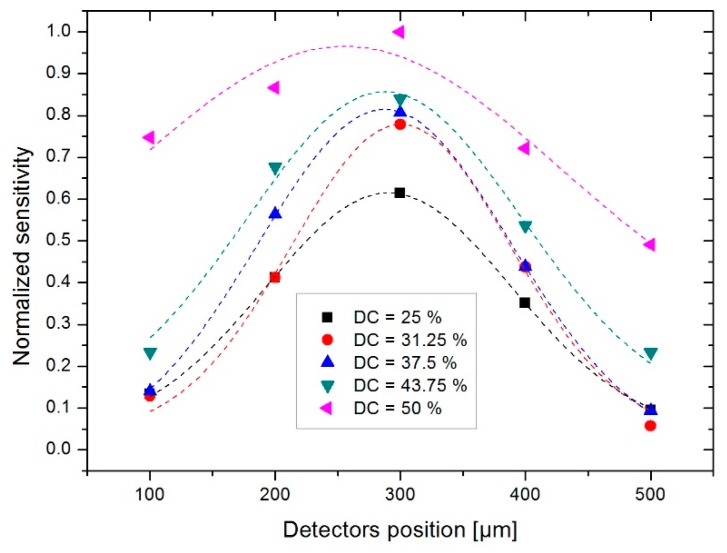
Evolution of the normalized sensitivity as a function of the position of detectors for duty cycles studied.

It is therefore of interest to study the evolution of sensitivity according to the position of the detectors. In Figure 17, the normalized sensitivity obtained for various duty cycles and a measurement range of ±300°/s, as a function of the position of detectors is recorded. In this figure, one notes that the optimal position to obtain the highest level of sensitivity is a distance of 300 µm between the heaters and the detectors, irrelevant of the duty cycle. This position has therefore been used for experimental development of the device.

### 4.6. Bandwidth Determination with N_2_ Medium

**Figure 18:**
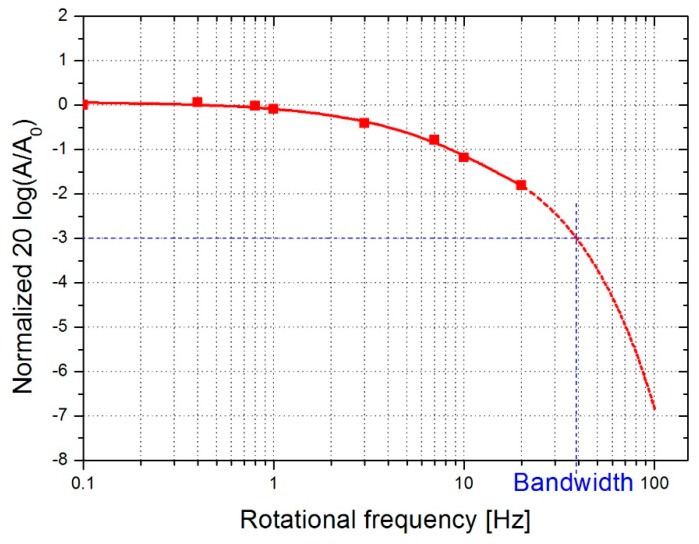
Frequency response of fabricated gyroscope.

The gyroscope bandwidth is an important characteristic of its operation. To carry out this study, a specific program has been developed in order to control the rate table. The latter was stable up to a rotational frequency of 20 Hz. The gyroscope has been tested at 18 mW power, 50% duty cycle and with N_2_ medium. The peak-to-peak amplitude A was normalized to the low frequency amplitude A_0_. Extrapolation up to 100 Hz has predicted a bandwidth of the order of 40 Hz, as shown in Figure 18. This result is of the same order of magnitude as that found in the literature [11], while knowing that we are not in the same geometrical configurations (size, distance and position of the heaters relative to the temperature detectors).

### 4.7. Shock Resistance

Since the design of a sensor without proof mass is considered to be potentially more resistant against mechanical shocks we felt obliged to perform corresponding tests. The idea is to submit an already characterized sensor to a high amplitude mechanical shock and to check it afterwards. This test enables, not only to define the mechanical resistance of the gyroscope, more particularly, that of the suspended bridges. As the prototype of the readout electronics required for this type of sensor is at present too cumbersome, the study was carried out exclusively to test the shock resistance of the gyroscope on its PCB support. 

**Figure 19:**
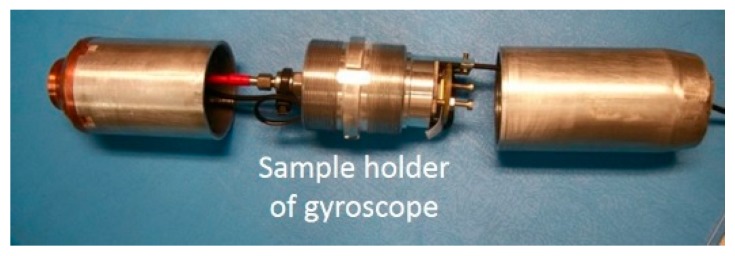
Test vehicle accelerated by electromagnetic force to generate high shocks.

The shock test was carried out using an electromagnetic accelerator, which drives a test vehicle. The working principle is based on the creation of a high-intensity magnetic field which is used to propel the vehicle at a controlled acceleration (amplitude and time) without the need for any mechanical transmission. The vehicle contains the gyroscope (Figure 19), of which the suspended bridge plane is placed normal to the acceleration axis which corresponds to the most sensitive axis from a mechanical point of view.

**Figure 20:**
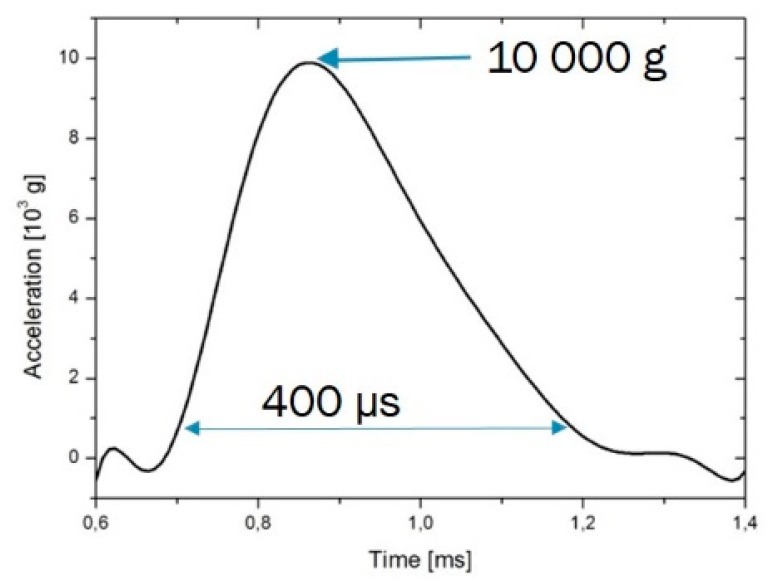
Acceleration phase of the vehicle during the shock test, as measured by a reference accelerometer in the vehicle. The maximum acceleration attained is around 10,000 g.

**Figure 21:**
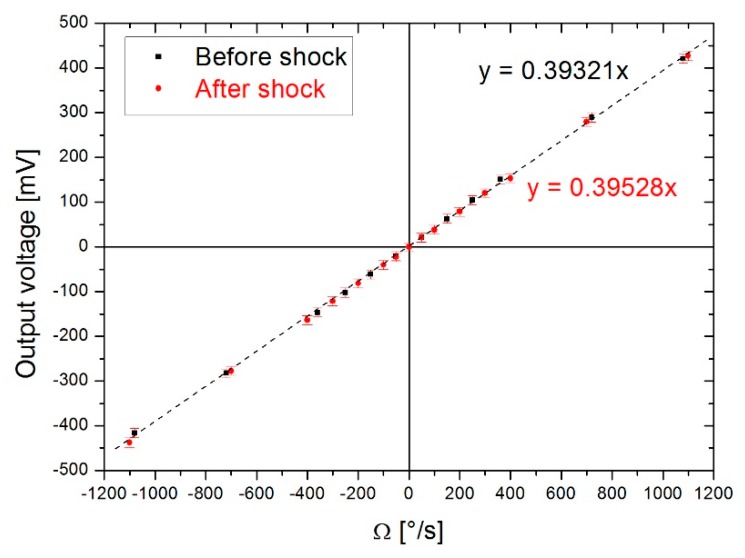
Sensors output voltage vs Ω measured before (**black**) & after (**red**) the test shock.

The gyroscope was subjected to a peak acceleration of around 10,000gees for a duration of 400 µs, as illustrated in Figure 20. No package legs, suspended bridges or soldered connections were torn off or damaged. The sensor therefore resisted mechanically. The characterization of the gyroscope after the shock test was then carried out under exactly the same conditions as before the shock test. Figure 21 indicates the gyroscope output as a function of the rotational velocity for measurements before and after the shock test. As shown in this figure, the gyroscope characteristics was preserved after having been subjected to the shock. Thanks to the mechanical resistance and the conservation of the gyroscopic measurements over a measurement range of ±1080°/s, the robustness of the prototype produced has been validated for a shock value of 10,000g over a duration of 400 µs. To our knowledge, no commercial MEMS gyroscope with such a measurement range, withstands such large shocks. Only few commercial gyroscopes withstand shocks of 10,000g but have a measurement ranges of only 300°/s.

## 5. Conclusions

The present study was carried out on a single-axis, gas thermal gyroscope using the thermal expansion phenomenon to generate flow within a cavity. The working principle requires alternating activation of two suspended micro-heaters used to heat a gas encapsulated inside a cavity provoking expansion of this gas. The hot gas stream is deflected due to rotation of the system. Two temperature detectors are placed symmetrically in the cavity to detect a temperature difference which is zero when the system is stationary. During rotation, the gas flow is deflected due to the Coriolis acceleration. Consequently, a temperature difference is recorded between the two detectors. This difference is in a linear relationship to the angular velocity. The angular velocity detection method has been proven both experimentally and numerically. 

These micro-sensors are manufactured using microelectronic technology to produce a simple structure which has no mobile solid proof mass, which enables them to resist against high shock levels. Indeed, the robustness of the prototype was validated in a shock test at 10,000 gees. Afterwards, gyroscopic measurements over an operating range of ±1080°/s have been successfully performed. Various parameters such as duty cycle, power injected into the heaters and the type of the gas have been studied with the aim to improve the sensitivity of the sensor. In this way, optimal operation, from a sensitivity viewpoint, corresponds to the use of SF_6_ as working gas, a heating duty cycle of 37.5%, a frequency of 32 Hz and a heating power of 18 mW. The sensor illustrates a sensibility of 0.0184 mK/(°/s) with a non-linearity of 0.1% over a measurement range of 1080°/s. 

In future, we intend to continue the characterization of the prototype by measuring the bandwidth with the gases used so far and to begin stability studies. The development of more compact readout electronics with appropriate shock resistance is envisaged. Moreover, the numerical simulation model must be improved in order to match our experimental results. This will in turn help to subsequently improve the characteristics of the device by numerical means.
